# A study of passive weight-bearing lower limb exercise effects on local muscles and whole body oxidative metabolism: a comparison with simulated horse riding, bicycle, and walking exercise

**DOI:** 10.1186/1476-5918-8-4

**Published:** 2009-11-10

**Authors:** Kohsuke Shimomura, Norio Murase, Takuya Osada, Ryotaro Kime, Mikiko Anjo, Kazuki Esaki, Kiyoshi Shiroishi, Takafumi Hamaoka, Toshihito Katsumura

**Affiliations:** 1 Department of Sports Medicine for Health Promotion, Tokyo Medical University, 6-1-1, Shinjuku, Shinjuku-ku Tokyo 160-8402, Japan; 2 Department of Judotherapy and Sports Medicine, Faculty of Health Science, Ryotokuji University, 5-8-1, Meikai, Urayasu, Chiba, 279-8567, Japan; 3 National Institute of Fitness and Sports in Kanoya, 1 Shiromizu, Kanoya, Kagoshima 891-2393, Japan

## Abstract

**Background:**

We have developed an exercise machine prototype for increasing exercise intensity by means of passively exercising lower limb muscles. The purpose of the present study was to compare the passive exercise intensity of our newly-developed machine with the intensities of different types of exercises. We also attempted to measure muscle activity to study how these forms of exercise affected individual parts of the body.

**Methods:**

Subjects were 14 healthy men with the following demographics: age 30 years, height 171.5 cm, weight 68.3 kg. They performed 4 types of exercise: Passive weight-bearing lower limb exercise (PWLLE), Simulated horse riding exercise (SHRE), Bicycle exercise, and Walking exercise, as described below at an interval of one week or longer. Oxygen uptake, blood pressure, heart rate, and electromyogram (EMG) were measured or recorded during exercise. At rest prior to exercise and immediately after the end of each exercise intensity, the oxygenated hemoglobin levels of the lower limb muscles were measured by near-infrared spectroscopy to calculate the rate of decline. This rate of decline was obtained immediately after exercise as well as at rest to calculate oxygen consumption of the lower limb muscles as expressed as a ratio of a post-exercise rate of decline to a resting one.

**Results:**

The heart rate and oxygen uptake observed in PWLLE during maximal intensity were comparable to that of a 20-watt bicycle exercise or 2 km/hr walking exercise. Maximal intensity PWLLE was found to provoke muscle activity comparable to an 80-watt bicycle or 6 km/hr walking exercise. As was the case with the EMG results, during maximal intensity PWLLE, the rectus femoris muscle consumed oxygen in amounts identical to that of an 80-watt bicycle or a 6 km/hr walking exercise.

**Conclusion:**

Passive weight-bearing lower limb exercise using our trial machine could provide approximately 3 MET of exercise and the thigh exhibited muscle activity equivalent to that of 80-watt bicycle or 6 km/hr walking exercise. Namely, given the same oxygen uptake, PWLLE exceeded bicycle or walking exercise in muscle activity, thus PWLLE is believed to strengthen muscle power while reducing the load imposed on the cardiopulmonary system.

## Background

It is widely recognized that appropriate exercise is effective for health promotion and prevents lifestyle-related diseases. Cycling and walking are conducted as popular forms of exercise for health promotion. Exercise can be more effective if the exercise intensity is appropriately determined by exercise testing. Some randomized-control trials showed that lifestyle modification has almost the same effects on aerobic capacity, body composition, and coronary risk factors as planned exercise programs for people who do not conduct daily exercise [1,2]. Under the conditions of the same amount of calories being expended daily, repetitive types of moderate exercise such as brisk walking, housework, and yard work are reportedly as effective as performing high intensity exercise [3,4]. Therefore, for health promotion, people should be encouraged to increase daily physical activity levels including frequency, intensity, duration, and exercise type, irrespective of training plans. Most patients with dyslipidemia, impaired glucose tolerance, or hypertension in need of exercise have symptoms such as gonalgia and lumbago that may discourage them from exercising, so they are prevented from practicing effective exercise.

Recently, various types of passive exercise equipment have been invented for health promotion and were created to provide incentive to exercise [5]. We define "passive weight-bearing exercise" in this study as that which trainees will conduct by motorized movements, but muscles are expected to be contract. This form of exercise may increase daily physical activity and may thereby help people get accustomed to conducting exercise because it can be done at any time, for any length, regardless of the weather, and without going to a gym. First, we developed an innovative stimulated horse riding machine that imitates the passive movements of bending forward and straightening up during horse riding. Simulated horse riding exercise (SHRE) might be useful as auxiliary therapy for the treatment of insulin resistance in type 2 diabetes [6]. However, during the developmental stage, in order to increase muscular strength primarily in the waist and back, the motion was designed using an electromyogram for effective discharge of erector spinae muscles. Therefore, its function was not sufficient for exercise intensity. Next, we developed a passive exercise machine prototype for increasing exercise intensity by means of passively exercising lower limb muscles [7]. Exercise using this machine is passive: the rider is compelled to lose his or her balance by an external force pressed against him or her; postural reflexes are provoked, prompting the rider to contract the lower limb muscles. In passive weight-bearing lower limb exercise (PWLLE), it was found that heart disease patients could tolerate exercise at an intensity of 70% of the anaerobic threshold. Because the cardiopulmonary function was thus not overloaded by this equipment, it might be of use in cardiac rehabilitation programs for heart disease patients [8]. However, the exercise intensity is uncertain and may be insufficient for health promotion.

The purpose of the present study was to compare the passive exercise intensity of our newly-developed machine with the intensities of SHRE and to clarify the loaded intensity on the PWLLE relative to cycling exercise and walking exercise. We also attempted to measure muscle activity in the abdomen, back, thigh, and leg, and to examine muscle metabolism in the thigh and leg. We thus tried to determine whether PWLLE affects lower-limb and whole-body oxidative metabolism.

## Methods

### Subjects

Subjects were 14 healthy men with the following demographics: age 30 ± 5 years, height 171.5 ± 5.0 cm, weight 68.3 ± 5.5 kg (mean ± SD). The study protocol was approved by the Tokyo Medical University Ethical Committee (approval number: 779) and the contents of the experiment were fully explained in writing to the subjects, who thereafter provided consent for participation in this study.

They performed 4 types of exercise as described below at an interval of one week or longer. Exercise devices as well as exercise protocols are described below.

### Exercise

#### Passive weight-bearing lower limb exercise (PWLLE)

**Figure 1:**
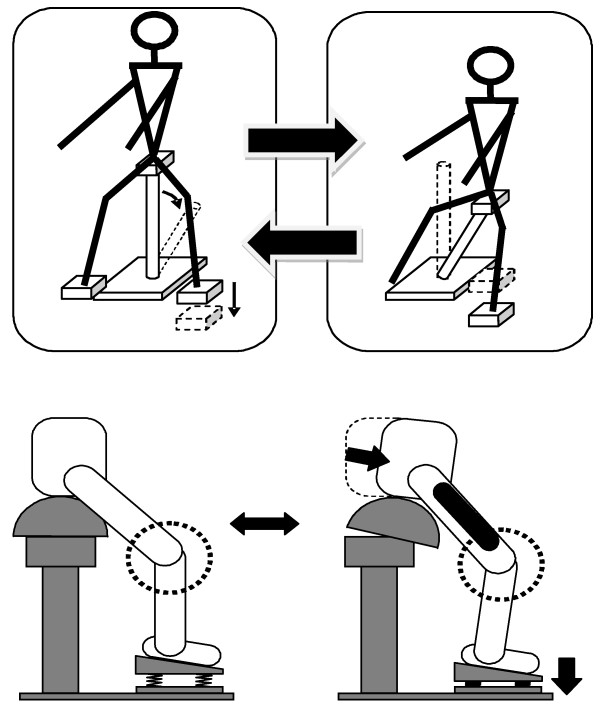
**Passive weight-bearing lower limb exercise equipment**. The equipment was designed so that the foot plates moved downward along with the inclining support rod. Therefore, the distance between the saddle and the foot plate could be kept constant and the subject could exercise without changing the knee joint angle. The subject had to try to balance the center-of-gravity shifts caused by the support rod that repetitively inclined alternatively on the left or right side, thus being more heavily loaded in the lower limb on the inclining side of the rod. The exercise intensity could be changed by altering the inclining movement cycle.

We devised a prototype machine to passively exercise the lower limbs (Panasonic Electric Works Co.) [7]. Its operation characteristics are shown in Figure 1. This equipment was composed of a saddle on which a subject sat, a rod to support the saddle, and two foot plates attached at the oblique front position to mount the feet. The saddle was adjustable for height so that the subject could do half-loaded exercise by keeping the flexion angle of the knee constant. The body weight of the subject was thus supported at three points by the saddle and both foot plates. The device induced motorized movements that moved the saddle repetitively in the front oblique direction.

**Figure 2:**
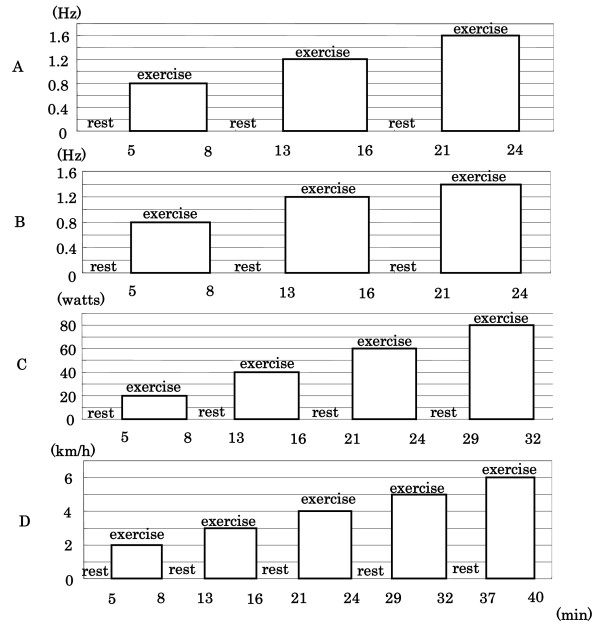
**Exercise protocols**. Exercise was conducted for 3 minutes at each of the exercise intensities, followed by a 5-minute rest. Blood pressure was monitored from 1 minute before the end of exercise. At the end of exercise, the arterial blood flow was occluded (by a pressure of 600 mmHg) for 1 minute at the proximal region of the left thigh. The exercise intensities were set as follows. A: passive weight-bearing lower limb exercise: 0.8 Hz, 1.2 Hz, and 1.6 Hz B: horse-ride-simulated exercise: 0.8 Hz, 1.2 Hz, and 1.4 Hz C: bicycle exercise: 20 watts, 40 watts, 60 watts, and 80 watts, D: walking exercise: 2 km/hr, 3 km/hr, 4 km/hr, 5 km/hr, and 6 km/hr

Moreover, in order to reduce pain associated with knee joint motion that might occur during exercise, the foot plates were designed to move downward in harmony with the support rod motion, which allowed the subject to do exercise while maintaining the knee joint angle because the distance between the saddle and foot plates was constant. Repeated alternate right or left side shifts of the subject's center of gravity caused by oblique movements of the support rod imposed a larger amount of load on the lower limbs on the side of the slanted rod because the limbs were mobilized to regain body balance. The exercise intensity could be changed by varying the slant cycles. Subjects were tasked with performing passive exercise using the prototype machine at three intensities of 0.8, 1.2, and 1.6 Hz for 3 minutes each with a 5-minute rest between performances (Figure 2A).

#### Simulated horse riding exercise (SHRE)

Commercially-available fitness equipment, SHRE (Panasonic Electric Works Co.) was used [5]. This equipment was designed to simulate the movements of horseback riding. To be more precise, the seat moved forward and backward periodically in order to passively exercise the trunk as well as the thighs of the subject. The exercise intensity could be adjusted by changing the motion cycle to 0.8, 1.2, and 1.4 Hz. These three cycles were imposed for a 3-minute exercise with a 5-minute rest between exercises (Figure 2B).

#### Bicycle exercise

A bicycle ergometer (RICO500, Lode Co., Netherlands) was used to conduct bicycle exercise at four intensities of 20, 40, 60, and 80 watts for 3 minutes each with a 5-minute rest between exercises (Figure 2C). The wheel rotation speed of each bicycle exercise was set at 50 revolutions per minute.

#### Walking exercise

A treadmill (MAT2600, Fukuda Denshi) was used for walking exercise, which was performed at five intensities of 2, 3, 4, 5, and 6 km/hr for 3 minutes with inter-exercise rests of 5 minutes (Figure 2D). Each subject was allowed to walk naturally at the same speed as the treadmill without restrictions on stride length or number of steps.

### Measurement items

Oxygen uptake (AERO MONITOR AE 300S, Minato Medical Science co., ltd), blood pressure, heart rate, and electromyogram (EMG) were measured or recorded during exercise. Blood pressure was measured at rest and at one minute before the end of exercise, while oxygen uptake used the breath-by-breath method. EMGs were recorded at four muscles: the right rectus femoris, right gastrocnemius, right rectus abdominis, and right erector spinae muscles. Electrodes were attached to the belly of the former two muscles 4 cm apart, while electrodes were attached both at the navel level and 4 cm cranial from it in the right rectus femoris muscle and both at the L4 level and 4 cm cranial from it in the right erector spinae muscle.

Resting maximal voluntary contraction strength (MVC) was also measured in each examined muscle. Measurements were taken once a day in each target muscle on four different days as follows. In the rectus femoris muscle, a custom-made knee-extending machine was used to extend the knee via isometric conditions of a subject in the sitting position as EMG was recorded (The angle of the knee joint was 90°). The mean of measured values of EMG was considered as MVC, and this procedure was applied to the other target muscles. MVC of the gastrocnemius muscle was obtained as follows: in the standing position, subjects tried to lift an immovable post over the head by standing on their tiptoes (The angle of the knee joint was 0° and the angle of the foot joint was 90°).

As for the erector spinae muscle, EMG was recorded while the subject tried to extend the trunk via isometric conditions from the sitting position with the trunk bent forward at a 90° angle. Concerning the rectus abdominis muscle, a custom-made machine was employed to let the subject move the trunk forward in the sitting position (The angle between trunk and hip joint was 90°).

EMG waveforms acquired from examined muscles were rectified and integrated to calculate integral EMG values (iEMG), ratios of which MVC as expressed as a percentage were then calculated for each muscle concerned.

**Figure 3:**
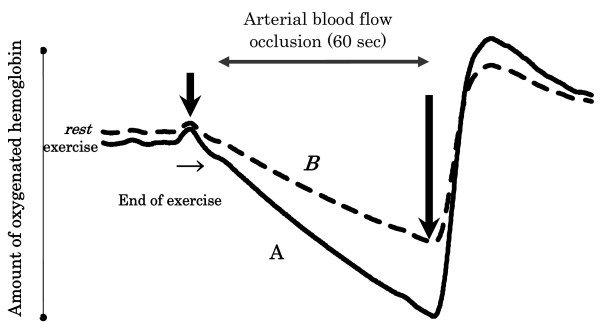
**Method of assessment of muscle oxygen consumption by near-infrared spectroscopy**. Measured at the left rectus femoris and gastrocnemius muscles. At rest and immediately after the end of exercise at each intensity, the proximal part of the leftf emoral region was occluded at 600 mmHg for 1 minute, during which oxygenated hemoglobin levels were acquired. "A" denotes a slope of oxygenated hemoglobin levels acquired at exercise, while "B" denotes that at rest. A value of A/B represents a ratio of oxygen consumed during exercise to that consumed at rest

At rest prior to exercise and immediately after the end of each exercise intensity, a pressure of 600 mmHg was applied to the proximal end of the left thigh for one minute to block arterial flow, during which time oxygenated hemoglobin levels were measured by near-infrared spectroscopy (Hamamatsu NIRO-200) to calculate the rate of decline. This rate of decline was obtained immediately after exercise as well as at rest to calculate the relative muscle metabolic rate of the lower limb muscles (photo detectors were attached to the left rectus femoris and left gastrocnemius muscles) as expressed as a ratio of a post-exercise rate of decline to a resting one (Figure 3).

### Statistical analysis

The differences of all parameters measured in this study in PWLLE and SHRE across exercise intensity were statistically analyzed by two-way ANOVA. Post hoc analyses were conducted with a paired t-test using SPSS (ver. 15). A significant p value was considered as less than 5%. All parameters in PWLLE at maximum intensity were compared to that of bicycle and walking exercise at each intensity and the nearest value was found. In order to compare individual differences (dissemination) at each exercise intensity between various forms of exercise, coefficients of variance were calculated. The coefficients of variance were calculated based on individual oxygen uptake values acquired by analysis of expired gas at each exercise intensity on different days.

## Results

Table 1 shows the results of heart rate, systolic blood pressure, and oxygen uptake measured for each exercise and at each intensity. Comparisons were made between stimulated horse riding exercise (SHRE) and passive weight-bearing lower limb exercise (PWLLE) at their maximal intensities, 1.4 and 1.6 Hz, respectively: heart rate 72.6 ± 5.2 bpm (SHRE) vs. 74.0 ± 2.9 bpm (PWLLE) (main effect NS, interaction NS), systolic blood pressure 135.7 ± 3.0 mmHg vs. 141.6 ± 4.2 mmHg (main effect NS, interaction NS), and oxygen uptake 6.9 ± 0.3 ml/kg/min vs. 10.0 ± 0.8 ml/kg/min (main effect p < 0.001, interaction p < 0.001), showing a significantly higher exercise intensity in PWLLE compared to SHRE when conducted at their maximal intensities (P < 0.001). The heart rate and oxygen uptake observed in PWLLE of the maximal intensity were comparable to that of a 20-watt bicycle exercise or 2 km/hr walking exercise.

**Table 1:** Heart rate (HR), systolic blood pressure (SBP), and oxygen uptake () for each form of exercise

	Intensity	HR (bpm)	SBP (mmHg)	(ml/kg/min)
**Passive weight-bearing lower limb exercise**	0.8 Hz	74.0 ± 2.88	135.5 ± 3.01	6.43 ± 0.29***
	
	1.2 Hz	77.4 ± 2.74	135.8 ± 3.99	8.04 ± 0.54***
	
	1.6 Hz	83.6 ± 3.12	141.6 ± 4.20	9.97 ± 0.80***

**Simulated horse riding exercise**	0.8 Hz	72.6 ± 5.20	131.0 ± 3.21	5.10 ± 0.28
	
	1.2 Hz	75.5 ± 4.84	131.7 ± 3.31	5.90 ± 0.29
	
	1.4 Hz	76.2 ± 4.73	135.7 ± 2.86	6.92 ± 0.33

**Bicycle exercise**	20 watts	81.0 ± 4.31	126.1 ± 5.05	9.85 ± 0.35 #
	
	40 watts	88.3 ± 4.17	129.7 ± 5.68	12.3 ± 0.34
	
	60 watts	95.6 ± 4.22	134.0 ± 5.85	15.6 ± 0.61
	
	80 watts	103.6 ± 4.56	138.8 ± 6.17	19.3 ± 0.61

**Walking exercise**	2 km/h	79.2 ± 3.47	135.2 ± 4.86	9.60 ± 0.34 #
	
	3 km/h	82.9 ± 3.11	136.7 ± 4.58	11.1 ± 0.38
	
	4 km/h	88.0 ± 2.98	138.5 ± 5.58	13.2 ± 0.50
	
	5 km/h	95.2 ± 2.96	144.7 ± 6.44	16.5 ± 0.73
	
	6 km/h	104.7 ± 3.52	148.3 ± 7.11	20.8 ± 0.70

				**(Mean ± SE)**

*P < 0.05, **P < 0.01, ***p < 0.001, simulated horse riding exercise (SHRE) vs passive weigt-bearing lower limb exercise (PWLLE) (0.8 Hz vs 0.8 Hz, 1.2 Hz vs 1.2 Hz, 1.4 Hz vs 1.6 Hz)

#The nearest  of PWLLE (1.6 Hz)

The results of muscle activity as measured in each exercise form by EMG are shown in Table 2 with regard to the rectus femoris, gastrocnemius, rectus abdominis, and erector spinae muscles. Maximal intensity PWLLE was found to provoke muscle activity comparable to an 80-watt bicycle or 6 km/hr walking exercise. Significantly higher muscle activity of the rectus femoris muscle was observed in PWLLE over SHRE at all of the intensities (SHRE vs. PWLLE; 0.8 Hz vs. 0.8 Hz, 1.2 Hz vs. 1.2 Hz, and 1.4 Hz vs. 1.6 Hz) (main effect p < 0.001, interaction p < 0.001). The results of relative muscle metabolic rate as measured in each form of exercise by near-infrared spectroscopy are shown in Table 3 concerning the rectus femoris as well as the gastrocnemius muscles. As was the case with the EMG results, in maximal intensity PWLLE, the rectus femoris muscle consumed oxygen in amounts identical to that of an 80-watt bicycle or a 6 km/hr walking exercise. Compared to SHRE, the rectus femoris muscle consumed significantly more oxygen at all intensities of PWLLE (main effect p < 0.001, interaction p < 0.001), while the gastrocnemius muscle did so only at the maximal intensity.

**Table 2:** Results of electromyographic evaluation of various muscles by exercise forms

	Intensity	Rectus Femoris	Gastrocnemius	Rectus abdominis	Erector spinae
**Passive weight-bearing lower limb exercise**	0.8 Hz	2.14 ± 0.43 *	1.09 ± 0.13	1.89 ± 0.48	1.64 ± 0.13
	
	1.2 Hz	3.33 ± 0.65**	1.45 ± 0.19	2.16 ± 0.61	1.93 ± 0.19
	
	1.6 Hz	4.63 ± 0.69	1.90 ± 0.22	2.69 ± 0.76	2.82 ± 0.36

**Simulated horse riding exercise**	0.8 Hz	1.08 ± 0.41	0.75 ± 0.07	1.58 ± 0.18	2.13 ± 0.54
	
	1.2 Hz	1.29 ± 0.33	1.15 ± 0.20	1.91 ± 0.18	2.47 ± 0.38
	
	1.4 Hz	1.55 ± 0.19	1.27 ± 0.25	1.99 ± 0.29	2.77 ± 0.42

**Bicycle exercise**	20 watts	2.62 ± 0.65	5.85 ± 1.34	1.59 ± 0.10	2.11 ± 0.30
	
	40 watts	3.50 ± 0.76	5.82 ± 1.25	1.83 ± 0.23	2.79 ± 0.50
	
	60 watts	4.27 ± 0.88	5.88 ± 1.23	1.99 ± 0.20	3.36 ± 0.47
	
	80 watts	4.80 ± 1.11 #	6.07 ± 1.19	2.20 ± 0.38	4.19 ± 1.21

**Walking exercise**	2 km/h	2.04 ± 0.32	6.61 ± 1.01	1.62 ± 0.13	7.96 ± 0.92
	
	3 km/h	2.26 ± 0.40	7.30 ± 0.87	1.61 ± 0.12	8.12 ± 1.39
	
	4 km/h	2.95 ± 0.34	8.15 ± 1.09	1.82 ± 0.11	7.62 ± 0.59
	
	5 km/h	3.64 ± 0.49	9.19 ± 1.21	1.89 ± 0.15	8.13 ± 0.71
	
	6 km/h	5.15 ± 0.73 #	10.2 ± 1.51	2.18 ± 0.18	9.24 ± 0.91

**(Mean ± SE)** **(Unit: % max activation)**					

*P < 0.05, **P < 0.01, ***p < 0.001, simulated horse riding exercise (SHRE) vs passive weight-bearing lower limb exercise (PWLLE) (0.8 Hz vs 0.8 Hz, 1.2 Hz vs 1.2 Hz, 1.4 Hz vs 1.6 Hz)

#The nearest value of PWLLE (1.6 Hz) at rectus femoris

**Table 3:** Results of relative muscle metabolic rate by exercise forms

	Intensity	Rectus femoris	Gastrocnemius
**Passive weight-bearing lower limb exercise**	0.8 Hz	2.13 ± 0.15**	1.13 ± 0.03
	
	1.2 Hz	2.78 ± 0.19***	1.32 ± 0.06
	
	1.6 Hz	4.05 ± 0.41***	1.74 ± 0.10*

**mulated horse riding exercise**	0.8 Hz	1.26 ± 0.07	1.10 ± 0.04
	
	1.2 Hz	1.57 ± 0.09	1.13 ± 0.10
	
	1.4 Hz	1.80 ± 0.18	1.23 ± 0.11

**Bicycle exercise**	20 watts	1.72 ± 0.13	1.50 ± 0.09
	
	40 watts	2.26 ± 0.21	2.03 ± 0.29
	
	60 watts	3.09 ± 0.29	2.60 ± 0.34
	
	80 watts	4.12 ± 0.37#	3.03 ± 0.39

**Walking exercise**	2 km/h	1.60 ± 0.42	2.71 ± 0.38
	
	3 km/h	2.03 ± 0.44	3.61 ± 0.39
	
	4 km/h	2.66 ± 0.42	4.30 ± 0.32
	
	5 km/h	3.35 ± 0.56	4.93 ± 0.48
	
	6 km/h	4.14 ± 0.78#	5.97 ± 0.44

**(Mean ± SE)** **(Unit: fold of resting)**			

*P < 0.05, **P < 0.01, ***p < 0.001 simulated horse riding exercise (SHRE) vs passive weight-bearing lower limb exercise (PWLLE)(0.8 Hz vs 0.8 Hz, 1.2 Hz vs 1.2 Hz, 1.4 Hz vs 1.6 Hz)

# The nearest value of PWLLE (1.6 Hz) at rectus femoris

In this study, we calculated coefficients of variation of oxygen uptakes as indices of individual differences in various exercises of the same intensity. These coefficients were 0.12 to 0.21 in PWLLE, 0.12 to 0.14 in SHRE, 0.07 to 0.10 in bicycle exercise, and 0.08 to 0.11 in walking exercise.

## Discussion

In the current study, our newly-developed passive exercise machine prototype provided an exercise intensity of roughly 3 METs, whereas the conventional simulated horse riding fitness machine provided only 2 METs. We thus demonstrated that the additional exercise of the lower limbs attained by our equipment improved exercise intensity compared to conventional equipment-aided exercises simulating horse riding. The Japanese Ministry of Fitness, Labour and Welfare recommended exercise of 3 METs or more in the Exercise Guidelines 2006 for Fitness [9], which was demonstrated to be fulfilled by our trial machine.

Evaluation of lower limb muscle activity as measured by EMG revealed that PWLLE at the maximal intensity provoked muscle activity almost identical to that of bicycle exercise at 80 watts or walking exercise at 6 km/hr. Namely, given the same oxygen uptake, PWLLE exceeded bicycle or walking exercise in muscle activity, thus PWLLE is believed to strengthen muscle power while reducing the load imposed on the cardiopulmonary system. This exercise is also thought to be applicable for people with knee joint pain. Patients with osteoarthritis of the knee, a representative disease of knee joint pain, are encouraged to exercise the quadriceps femoris muscle as conservative therapy [10]. It is believed that this ergotherapy alleviates gonalgia early, resulting in relieving muscle contractions induced by pain, followed by a secondary increase of muscle strength. Our new equipment was especially designed to relieve the load on the knee joint, so even people with knee joint pain will be able to continue exercise. Considering the lower limb muscles, mainly the thigh, might be efficiently trained, it is promising that this training will lead to alleviation of knee joint pain itself.

Muscle oxygen dynamics in the lower limbs were assessed during exercise by near-infrared spectroscopy in this study. Muscle oxygenation levels have been reported in various forms of exercise such as bicycle exercise [11-13], knee joint extension exercise [14], and grip exercise [15]. There is a problem in comparative evaluation because muscle oxygenation levels cannot be compared easily between different individuals. In order to evaluate muscle metabolic rate, the transient arterial occlusion method was employed for correction in this study. The principle of oxygen consumption measurement resides in the fact that oxygen consumption is reflected by an initial declining rate of muscle oxygenation levels which are reduced by the interruption of oxygen supply to tissues [16].

Compared to SHRE, PWLLE augmented relative muscle metabolic rate of the femoris muscle and total oxygen uptake. While the increase in femoris muscle activation with the PWLLE device was relatively small, it might still be responsible for or contribute to the increase in whole body oxygen uptake seen with this device. Therefore, we have succeeded in increasing exercise intensity in terms of local muscle and whole body oxidative metabolism using PWLLE.

We evaluated variations of exercise intensities due to differences in exercise forms using coefficients of variation in this study. At similar total oxygen consumptions at 1.6 Hz for PLLE, 20-watt for bicycle exercise, 2 km/hr for walking exercise, and 1.4 Hz (at its maximum intensity) for SHRE, the coefficients were 0.21, 0.09, 0.09, and 0.12, respectively, clearly showing the largest coefficient for PWLLE. It was therefore clarified that PWLLE, at the intensity of 3METs, was most likely to cause individual differences at the same intensity among the four forms of exercise. Hence, this equipment needs to be improved in the areas of achievable and quantitative intensity, which are both inferior to walking as well as bicycle exercise, although its achievable intensity was the strongest among commercial fitness equipment.

Since our equipment can perform higher intensity exercise, more efficient exercise therapy is expected. In the future, we need to study further the effects of continuous exercise therapy using this equipment on people with a history of lifestyle-related diseases.

## Conclusion

Passive weight-bearing lower limb exercise using our trial machine could provide approximately 3 MET of exercise and the thigh exhibited muscle activity equivalent to that of 80-watt bicycle or 6 km/hr walking exercise. Namely, given the same oxygen uptake, PWLLE exceeded bicycle or walking exercise in muscle activity, thus PWLLE is believed to strengthen muscle power while reducing the load imposed on the cardiopulmonary system.

This equipment is easily accessible for indoor exercise, is safe and low in exercise intensity, does not impose a load on the knee joint, and enables one to train the lower limb, mainly thigh, muscles. This equipment might therefore be useful for exercise in elderly people, weak people, people with knee joint pain, and obese people.

## Competing interests

The authors declare that they have no competing interests. The machine we used in this study was rented from Panasonic Electric Works Co. However, this study was not performed for commercial purposes and the data from this study were not and will not be used for commercial purposes. This study was performed exclusively to evaluate properly whether or not passive exercise equipment provided an exercise form that was of help in improving habitual exercise.

## Authors' contributions

KS performed selection of the experiment subjects as well as the measurement and evaluation of NIRS data. NM served as the general administrator for the experiment and performed the cast immobilization process. TK and TO performed selection and medical checks of the experiment subjects. RK, KE, and KS (Shiroisi) performed measurement and evaluation of NIRS data. TK, MN, and TO examined medical checks of the experiment subjects. All authors read and approved the final manuscript.
